# In this month’s Bulletin

**DOI:** 10.2471/BLT.21.000321

**Published:** 2021-03-01

In the editorial section, Matthew Neilson et al. (170) call for improved quality of care in conflict-affected settings. M Anne Yu et al. (171) explain how WHO is coordinating the deployment of vaccines for COVID-19. 

In the news section, Tatum Anderson (174–175) reports on barriers and solutions to national vaccine deployments. Claire Wardle talks to Andréia Azevedo Soares (176–177) about the challenges faced in addressing false or misleading information about health.

## Bangladesh

### Reproductive health services for a refugee population

Md Nuruzzaman Khan et al. (201–208) track access to female contraceptives.

## Brazil, Cambodia, China, Congo, Egypt, India, Islamic Republic of Iran, Malaysia, Mexico, Mongolia, Pakistan, Philippines, South Africa, Turkey

### Telemedicine for diabetes 

Jorge César Correia et al. (209–219) review the evidence on effective delivery of services.

## Mexico

### Maternal health services 

Edson Serván-Mori et al. (190–200) assess the continuum of care

## India

### An essential diagnostics list

Sonam Vijay et al. (236–238) recount the process for introduction. 

## South Africa

### Caring for mothers and babies

Tsakane MAG Hlongwane et al. (236–238) study implementation of antenatal care recommendations.

## United Kingdom of Great Britain and Northern Ireland

### Early COVID-19 cases

Nicola L Boddington et al. (178–189) describe clinical and epidemiological characteristics.

## Global

### Who measures what for sustainable development? 

Fabrício Silveira et al. (228–235) calculate convergence on indicators.

### Tracking antimicrobial resistance

Christine Årdal et al. (239–240) propose using the system for environmental surveillance of polio.

